# Poly[bis­[μ_3_-2-(1*H*-tetra­zol-1-yl)acetato]cadmium(II)]

**DOI:** 10.1107/S160053680904255X

**Published:** 2009-10-23

**Authors:** Li-Xia Xie, Xian-Fu Zheng, Hui Su, Qiu Jin

**Affiliations:** a College of Sciences, Henan Agricultural University, Zhengzhou, Henan 450002, People’s Republic of China

## Abstract

In the title compound, [Cd(C_3_H_3_N_4_O_2_)_2_]_*n*_, the Cd^II^ ion, located on a twofold rotation axis, is six-coordinated by two N atoms [Cd—N = 2.368 (2) Å] and four O atoms [Cd—O = 2.300 (1) and 2.260 (1) Å] from six 2-(1*H*-tetra­zol-1-yl)acetate (*L*) ligands in a distorted octa­hedral geometry. The metal centres are connected *via* the tridentate *L* ligands into a three-dimensional polymeric structure.

## Related literature

For related structures, see: Du *et al.* (2007 ▶); Lee *et al.* (2005 ▶); Won *et al.* (2007 ▶); Yang *et al.* (2009 ▶).
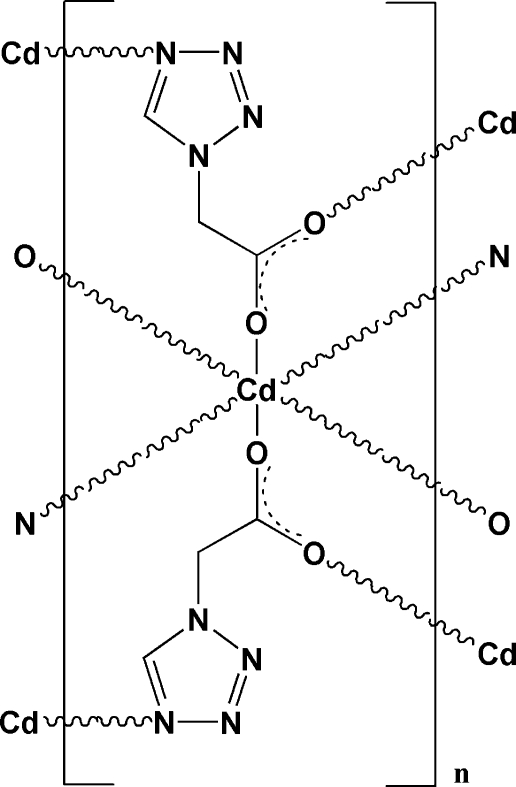


## Experimental

### 

#### Crystal data

[Cd(C_3_H_3_N_4_O_2_)_2_]
*M* *_r_* = 366.60
Monoclinic, 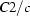

*a* = 14.750 (3) Å
*b* = 8.857 (2) Å
*c* = 9.503 (2) Å
β = 118.42 (3)°
*V* = 1091.8 (4) Å^3^
*Z* = 4
Mo *K*α radiation
μ = 2.03 mm^−1^
*T* = 293 K
0.20 × 0.20 × 0.20 mm

#### Data collection

Rigaku Mercury CCD diffractometer
Absorption correction: multi-scan (*CrystalClear*; Rigaku, 2000 ▶) *T*_min_ = 0.666, *T*_max_ = 0.673
6805 measured reflections
1452 independent reflections
1441 reflections with *I* > 2σ(*I*)
*R*_int_ = 0.025

#### Refinement

*R*[*F* ^2^ > 2σ(*F* ^2^)] = 0.017
*wR*(*F* ^2^) = 0.049
*S* = 0.94
1452 reflections
87 parameters
H-atom parameters constrained
Δρ_max_ = 0.38 e Å^−3^
Δρ_min_ = −0.44 e Å^−3^

### 

Data collection: *CrystalClear* (Rigaku, 2000 ▶); cell refinement: *CrystalClear*; data reduction: *CrystalClear*; program(s) used to solve structure: *SHELXS97* (Sheldrick, 2008 ▶); program(s) used to refine structure: *SHELXL97* (Sheldrick, 2008 ▶); molecular graphics: *SHELXTL* (Sheldrick, 2008 ▶); software used to prepare material for publication: *SHELXTL*.

## Supplementary Material

Crystal structure: contains datablocks global, I. DOI: 10.1107/S160053680904255X/cv2629sup1.cif

Structure factors: contains datablocks I. DOI: 10.1107/S160053680904255X/cv2629Isup2.hkl

Additional supplementary materials:  crystallographic information; 3D view; checkCIF report

## Figures and Tables

**Fig. 1.**
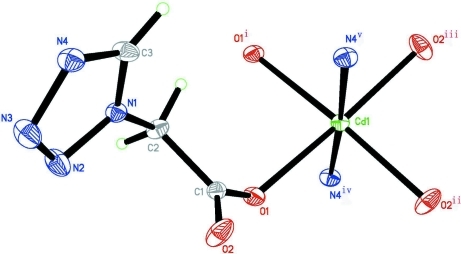
A portion of the crystal structure of (I) showing 30% probability displacement ellipsoids and the atomic labeling [symmetry codes: (i) - x, y, - z + 1/2; (ii) - x, - y + 1, - z; (iii) x, - y + 1, z + 1/2; (iv) x - 1/2, y + 1/2, z; (v) - x + 1/2, y + 1/2, - z + 1/2].

**Fig. 2.**
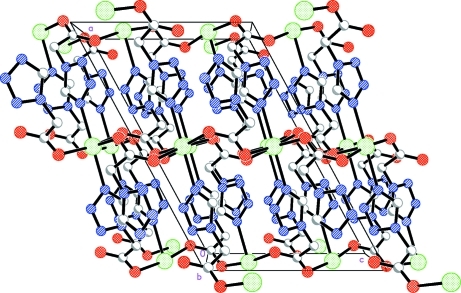
The crystal packing viewed along the b axis. H atoms omitted for clarity.

## supplementary crystallographic information

### Comment

Multidentate ligands containing rich coordination sites (N and/or O donors) are often employed to produce polymeric networks with structural diversity owing to their various coordination modes (Won *et al.*, 2007; Lee *et al.*, 2005; Du *et al.*, 2007). As ligands with multiple coordination site, tetrazole and its derivatives have been shown to be good organic linker in generation of structurally versatile metal-organic frameworks since it can bridge different metal centers to afford coordination polymers that exhibit extraordinary structural diversity and facile accessibility of functionalized materials (Yang *et al.*, 2009). Here, we report the synthesis and crystal structure of the title compound, (I).

In (I) (Fig. 1), each Cd^II^ ion located on a twofold rotation axis is six–coordinated by two tetrazole nitrogen atoms (N4) and four carboxylate group oxygen atoms (O1 and O2) from six distinct ligands. The coordination bond lengths Cd—N and Cd—O are 2.368 (2), 2.300 (1) Å and 2.260 (1) Å, respectively. The coordination geometry around Cd^II^ can be described as a distorted octahedron - the Cd^II^ coordination angles are in the range 83.34 (6)° - 177.16 (7)°. Each fully deprotonated *L* ligand serves as a tridentate bridging ligand *via* one nitrogen atom at the 5-position of the tetrazole ring while the nitrogen atoms at 3,4-positions remain uncoordinated, and two carboxylate O atoms. In this way two metal atoms and two carboxylate form a 8-membered [*M*_2_C_2_O_4_] metallocyclic ring, the Cd···Cd distance is 4.793 Å. The Cd···Cd distance linked by the bridged *L* ligand is 8.603 Å. Thus each Cd^II^ centers are linked together by six *L* ligands into a three–dimensional polymeric structure (Fig. 2).

### Experimental

All solvents and chemicals were of analytical grade and were used without further purification. The compound [Cd*L*_2_]_n_ was synthesized as follows: 2-(1H–tetrazol–1–yl) acetic acid (1.0 mmol) was added to 5 cm^3^ water and the resulting solution was adjusted pH to 7.0 by NaOH aqueous. Then Cd(NO_3_)_2_(0.5 mmol) was added to the above solution, and the mixture was stirred for 30 min and filtered. After two days, colourless single crystals suitable for X-ray analysis were obtained. Anal. Calcd (%) for C_6_H_6_CdN_8_O_4_: C, 19.66; H, 1.65; N, 30.57. Found (%): C, 19.79; H, 1.45; N, 30.46.

### Refinement

The H atoms were included in calculated positions and treated as riding atoms: C–H = 0.93 Å for the tetrazole and 0.97 Å for the methylene H atoms, with *U*_iso_(H) = 1.2*U*_eq_(C).

### Crystal data

**Table:**

[Cd(C_3_H_3_N_4_O_2_)_2_]	*F*(000) = 712
*M**_r_* = 366.60	*D*_x_ = 2.230 Mg m^−^^3^
Monoclinic, *C*2/*c*	Mo *K*α radiation, λ = 0.71073 Å
Hall symbol: -C 2yc	Cell parameters from 2016 reflections
*a* = 14.750 (3) Å	θ = 2.4–29.1°
*b* = 8.857 (2) Å	µ = 2.03 mm^−^^1^
*c* = 9.503 (2) Å	*T* = 293 K
β = 118.42 (3)°	Prism, colourless
*V* = 1091.8 (4) Å^3^	0.20 × 0.20 × 0.20 mm
*Z* = 4	

### Data collection

**Table:**

Rigaku Mercury CCD diffractometer	1452 independent reflections
Radiation source: fine-focus sealed tube	1441 reflections with *I* > 2σ(*I*)
graphite	*R*_int_ = 0.025
ω scans	θ_max_ = 29.1°, θ_min_ = 3.2°
Absorption correction: multi-scan (*CrystalClear*; Rigaku, 2000)	*h* = −20→19
*T*_min_ = 0.666, *T*_max_ = 0.673	*k* = −12→12
6805 measured reflections	*l* = −13→12

### Refinement

**Table:**

Refinement on *F*^2^	Primary atom site location: structure-invariant direct methods
Least-squares matrix: full	Secondary atom site location: difference Fourier map
*R*[*F*^2^ > 2σ(*F*^2^)] = 0.017	Hydrogen site location: inferred from neighbouring sites
*wR*(*F*^2^) = 0.049	H-atom parameters constrained
*S* = 0.94	*w* = 1/[σ^2^(*F*_o_^2^) + (0.0394*P*)^2^ + 0.8*P*] where *P* = (*F*_o_^2^ + 2*F*_c_^2^)/3
1452 reflections	(Δ/σ)_max_ = 0.001
87 parameters	Δρ_max_ = 0.38 e Å^−^^3^
0 restraints	Δρ_min_ = −0.44 e Å^−^^3^

### Special details

**Table:**

Geometry. Bond distances, angles *etc*. have been calculated using the rounded fractional coordinates. All su's are estimated from the variances of the (full) variance-covariance matrix. The cell e.s.d.'s are taken into account in the estimation of distances, angles and torsion angles
Refinement. Refinement of *F*^2^ against ALL reflections. The weighted *R*-factor *wR* and goodness of fit *S* are based on *F*^2^, conventional *R*-factors *R* are based on *F*, with *F* set to zero for negative *F*^2^. The threshold expression of *F*^2^ > σ(*F*^2^) is used only for calculating *R*-factors(gt) *etc*. and is not relevant to the choice of reflections for refinement. *R*-factors based on *F*^2^ are statistically about twice as large as those based on *F*, and *R*- factors based on ALL data will be even larger.

### Fractional atomic coordinates and isotropic or equivalent isotropic displacement parameters (Å^2^)

**Table:**

	*x*	*y*	*z*	*U*_iso_*/*U*_eq_	
Cd1	0.0000	0.535583 (15)	0.2500	0.01857 (7)	
O1	−0.03228 (9)	0.34712 (15)	0.06557 (14)	0.0254 (2)	
O2	0.05506 (13)	0.28052 (17)	−0.06267 (17)	0.0377 (3)	
N3	0.25046 (14)	−0.05131 (19)	0.0670 (2)	0.0335 (4)	
N4	0.32460 (13)	0.02896 (15)	0.1891 (2)	0.0257 (3)	
C3	0.27659 (12)	0.1113 (2)	0.24775 (19)	0.0260 (3)	
H3A	0.3077	0.1781	0.3332	0.031*	
N1	0.17630 (10)	0.08377 (16)	0.16511 (16)	0.0205 (2)	
C1	0.03356 (12)	0.27150 (17)	0.04894 (17)	0.0208 (3)	
C2	0.09014 (12)	0.15137 (19)	0.17684 (18)	0.0222 (3)	
H2A	0.1155	0.1969	0.2817	0.027*	
H2B	0.0418	0.0726	0.1669	0.027*	
N2	0.16106 (13)	−0.01883 (19)	0.0519 (2)	0.0329 (4)	

### Atomic displacement parameters (Å^2^)

**Table:**

	*U*^11^	*U*^22^	*U*^33^	*U*^12^	*U*^13^	*U*^23^
Cd1	0.01602 (10)	0.02182 (10)	0.01872 (10)	0.000	0.00896 (7)	0.000
O1	0.0187 (5)	0.0290 (6)	0.0235 (5)	0.0055 (4)	0.0058 (4)	−0.0047 (4)
O2	0.0526 (9)	0.0394 (7)	0.0324 (7)	0.0211 (6)	0.0295 (7)	0.0158 (6)
N3	0.0222 (8)	0.0369 (8)	0.0392 (9)	0.0018 (6)	0.0127 (7)	−0.0138 (6)
N4	0.0177 (7)	0.0329 (8)	0.0255 (7)	0.0025 (5)	0.0093 (6)	−0.0021 (5)
C3	0.0172 (7)	0.0354 (8)	0.0217 (7)	0.0015 (6)	0.0064 (6)	−0.0056 (6)
N1	0.0168 (6)	0.0234 (6)	0.0203 (6)	0.0022 (5)	0.0081 (5)	−0.0005 (5)
C1	0.0198 (7)	0.0219 (6)	0.0182 (6)	0.0018 (5)	0.0070 (5)	−0.0005 (5)
C2	0.0178 (6)	0.0298 (7)	0.0212 (7)	0.0061 (5)	0.0111 (5)	0.0048 (6)
N2	0.0222 (8)	0.0339 (8)	0.0397 (10)	−0.0015 (6)	0.0124 (7)	−0.0153 (7)

### Geometric parameters (Å, °)

**Table:**

Cd1—O2^i^	2.2595 (14)	N3—N4	1.356 (2)
Cd1—O2^ii^	2.2595 (14)	N4—C3	1.311 (2)
Cd1—O1	2.3000 (13)	N4—Cd1^vi^	2.3678 (17)
Cd1—O1^iii^	2.3000 (13)	C3—N1	1.326 (2)
Cd1—N4^iv^	2.3678 (17)	C3—H3A	0.9300
Cd1—N4^v^	2.3678 (17)	N1—N2	1.343 (2)
O1—C1	1.2502 (19)	N1—C2	1.4564 (19)
O2—C1	1.246 (2)	C1—C2	1.530 (2)
O2—Cd1^ii^	2.2595 (14)	C2—H2A	0.9700
N3—N2	1.289 (2)	C2—H2B	0.9700
			
O2^i^—Cd1—O2^ii^	87.75 (8)	C3—N4—Cd1^vi^	129.09 (12)
O2^i^—Cd1—O1	171.86 (5)	N3—N4—Cd1^vi^	124.37 (12)
O2^ii^—Cd1—O1	93.23 (6)	N4—C3—N1	108.77 (15)
O2^i^—Cd1—O1^iii^	93.23 (6)	N4—C3—H3A	125.6
O2^ii^—Cd1—O1^iii^	171.86 (5)	N1—C3—H3A	125.6
O1—Cd1—O1^iii^	86.94 (7)	C3—N1—N2	108.25 (14)
O2^i^—Cd1—N4^iv^	98.72 (6)	C3—N1—C2	130.32 (14)
O2^ii^—Cd1—N4^iv^	83.34 (6)	N2—N1—C2	121.34 (14)
O1—Cd1—N4^iv^	89.42 (5)	O2—C1—O1	126.95 (15)
O1^iii^—Cd1—N4^iv^	88.52 (5)	O2—C1—C2	117.22 (14)
O2^i^—Cd1—N4^v^	83.34 (6)	O1—C1—C2	115.79 (13)
O2^ii^—Cd1—N4^v^	98.72 (6)	N1—C2—C1	113.02 (12)
O1—Cd1—N4^v^	88.52 (5)	N1—C2—H2A	109.0
O1^iii^—Cd1—N4^v^	89.42 (5)	C1—C2—H2A	109.0
N4^iv^—Cd1—N4^v^	177.16 (7)	N1—C2—H2B	109.0
C1—O1—Cd1	126.41 (11)	C1—C2—H2B	109.0
C1—O2—Cd1^ii^	125.12 (11)	H2A—C2—H2B	107.8
N2—N3—N4	110.14 (16)	N3—N2—N1	106.81 (15)
C3—N4—N3	106.04 (15)		
			
O2^i^—Cd1—O1—C1	12.4 (4)	Cd1^ii^—O2—C1—O1	10.9 (3)
O2^ii^—Cd1—O1—C1	109.08 (14)	Cd1^ii^—O2—C1—C2	−171.73 (11)
O1^iii^—Cd1—O1—C1	−79.07 (13)	Cd1—O1—C1—O2	−108.22 (18)
N4^iv^—Cd1—O1—C1	−167.62 (14)	Cd1—O1—C1—C2	74.34 (18)
N4^v^—Cd1—O1—C1	10.43 (14)	C3—N1—C2—C1	100.0 (2)
N2—N3—N4—C3	0.1 (2)	N2—N1—C2—C1	−76.1 (2)
N2—N3—N4—Cd1^vi^	−172.37 (14)	O2—C1—C2—N1	11.3 (2)
N3—N4—C3—N1	−0.2 (2)	O1—C1—C2—N1	−171.02 (14)
Cd1^vi^—N4—C3—N1	171.87 (11)	N4—N3—N2—N1	−0.1 (2)
N4—C3—N1—N2	0.1 (2)	C3—N1—N2—N3	0.0 (2)
N4—C3—N1—C2	−176.36 (15)	C2—N1—N2—N3	176.83 (16)

Symmetry codes: (i) *x*, −*y*+1, *z*+1/2; (ii) −*x*, −*y*+1, −*z*; (iii) −*x*, *y*, −*z*+1/2; (iv) *x*−1/2, *y*+1/2, *z*; (v) −*x*+1/2, *y*+1/2, −*z*+1/2; (vi) *x*+1/2, *y*−1/2, *z*.
